# Spontaneous Tumor Lysis Syndrome in Refractory Metastatic Esophageal Adenocarcinoma Presenting With Septic Shock

**DOI:** 10.7759/cureus.115957

**Published:** 2026-09-08

**Authors:** Andrew T Ng, Marlee Thomas, Kevin T Jamouss, Abigail Orenstein

**Affiliations:** 1 Department of Internal Medicine, University of Massachusetts Chan Medical School - Baystate Regional Campus, Springfield, USA; 2 Department of Critical Care Medicine, University of Massachusetts Chan Medical School - Baystate Regional Campus, Springfield, USA

**Keywords:** acute renal failure, esophageal adenocarcinoma (eac), gi oncology, liver metastatic disease, medical icu, oncologic emergencies, solid tumor, supportive and palliative care, tumor lysis syndrome

## Abstract

Spontaneous tumor lysis syndrome (STLS) presents a diagnostic challenge when it occurs in patients with advanced solid malignancies and concurrent critical illness. We present a case of STLS in a 30-year-old man with refractory metastatic esophageal adenocarcinoma who presented with hypotension, anuria, and severe metabolic derangements. Laboratory evaluation demonstrated hyperuricemia (18.1 mg/dL), hyperkalemia (7.6 mmol/L), hyperphosphatemia (7.8 mg/dL), and acute kidney injury. Ionized calcium was also low at 1.03 mmol/L despite hypoalbuminemia, with laboratory and clinical findings consistent with TLS by Cairo-Bishop thresholds. His final treatment was initiated 19 days before presentation, with the continuous fluorouracil infusion completed approximately 17 days before presentation, placing the presentation well outside the conventional treatment-related timeframe. His course was complicated by septic shock secondary to methicillin-sensitive *Staphylococcus aureus* bacteremia. Despite supportive care and rasburicase administration, he developed multiorgan failure and was transitioned to hospice. This case highlights the rare occurrence of spontaneous TLS in metastatic esophageal adenocarcinoma and emphasizes the importance of early recognition and consideration in any patient with high tumor burden.

## Introduction

Tumor lysis syndrome (TLS) is an oncologic emergency caused by rapid tumor cell breakdown and release of intracellular contents into systemic circulation, resulting in hyperuricemia, hyperkalemia, hyperphosphatemia, and hypocalcemia [1]. These metabolic abnormalities can lead to acute kidney injury, cardiac arrhythmias, seizures, and ultimately death [1].

The Cairo-Bishop classification defines laboratory TLS as the presence of at least two metabolic abnormalities, including uric acid ≥8 mg/dL or a 25% increase from baseline, potassium ≥6.0 mmol/L or a 25% increase, phosphorus ≥4.5 mg/dL or a 25% increase, or corrected calcium ≤7.0 mg/dL or a 25% decrease, occurring within three days before or seven days after initiation of therapy [2]. It then defines clinical TLS as laboratory TLS with associated complications such as renal failure, arrhythmia, or seizure [2]. Spontaneous tumor lysis syndrome (STLS) is classically described as TLS occurring without a precipitating anticancer treatment; however, STLS has also been reported in patients with malignancy outside the conventional temporal relationship to treatment [1-5].

TLS most commonly occurs in hematologic malignancies following cytotoxic therapy [3]. In contrast, TLS in solid tumors is rare, and STLS, occurring without a clear temporal relationship to recent treatment, is even less common [3-5]. Although the Cairo-Bishop criteria incorporate a treatment-related timeframe, STLS has been reported outside of this window, particularly in patients with substantial tumor burden, high tumor cell turnover, elevated lactate dehydrogenase (LDH), renal dysfunction, and/or volume depletion [6]. Gastroesophageal adenocarcinoma represents an especially uncommon setting for STLS, with only isolated cases reported [4,5]. Thus, recognition of STLS in patients with gastroesophageal adenocarcinoma is especially challenging when patients are critically ill with competing diagnoses such as sepsis.

As anticancer therapeutics and diagnostic recognition improve, STLS in solid tumors is being reported with increasing frequency [7,8]. It tends to occur in aggressive, rapidly proliferating solid tumors with a large disease burden, frequently with hepatic metastatic disease [8]. In the intensive care unit (ICU), early recognition is paramount, as STLS can rapidly progress to severe acute kidney injury, fatal arrhythmias, and multiorgan failure. Supportive care must be initiated promptly, prioritizing appropriate intravenous (IV) hydration, electrolyte correction, and administration of hypouricemic agents such as rasburicase or allopurinol, with renal replacement therapy used for refractory metabolic derangements [6,7].

## Case presentation

A 30-year-old man with metastatic esophageal adenocarcinoma (HER2-amplified, Combined Positive Score of 15 for programmed death-ligand 1) with extensive liver metastases and bulky lymphadenopathy presented with generalized weakness, lethargy, and hypotension [9]. He had experienced progressive somnolence over five days, poor oral intake, and absence of urine output for one to two days prior to presentation.

His oncologic history included treatment with FOLFOX and trastuzumab administered every 14 days, with pembrolizumab 400 mg administered every six weeks. He received six biweekly administrations of FOLFOX and trastuzumab, with pembrolizumab administered twice during this period. His final treatment with FOLFOX, trastuzumab, and pembrolizumab was initiated 19 days before presentation, with the continuous fluorouracil infusion completed approximately 17 days before presentation. A subsequent FOLFOX and trastuzumab treatment was planned but was not administered before hospitalization. He had undergone the removal of 5 L of ascitic fluid three days prior to presentation for malignant ascites.

When he arrived at the hospital, he was hypotensive and required admission to the medical ICU for vasopressor support. Blood cultures were positive for methicillin-sensitive *Staphylococcus aureus* (MSSA). Contrast-enhanced computed tomography demonstrated extensive metastatic disease with innumerable hepatic metastases, bulky retroperitoneal and mediastinal lymphadenopathy, and ascites (Figure 1). Comparison with imaging obtained approximately two weeks earlier demonstrated interval progression of the metastatic disease. Pretreatment imaging done at an outside institution was unavailable for direct comparison.

**Figure 1:**
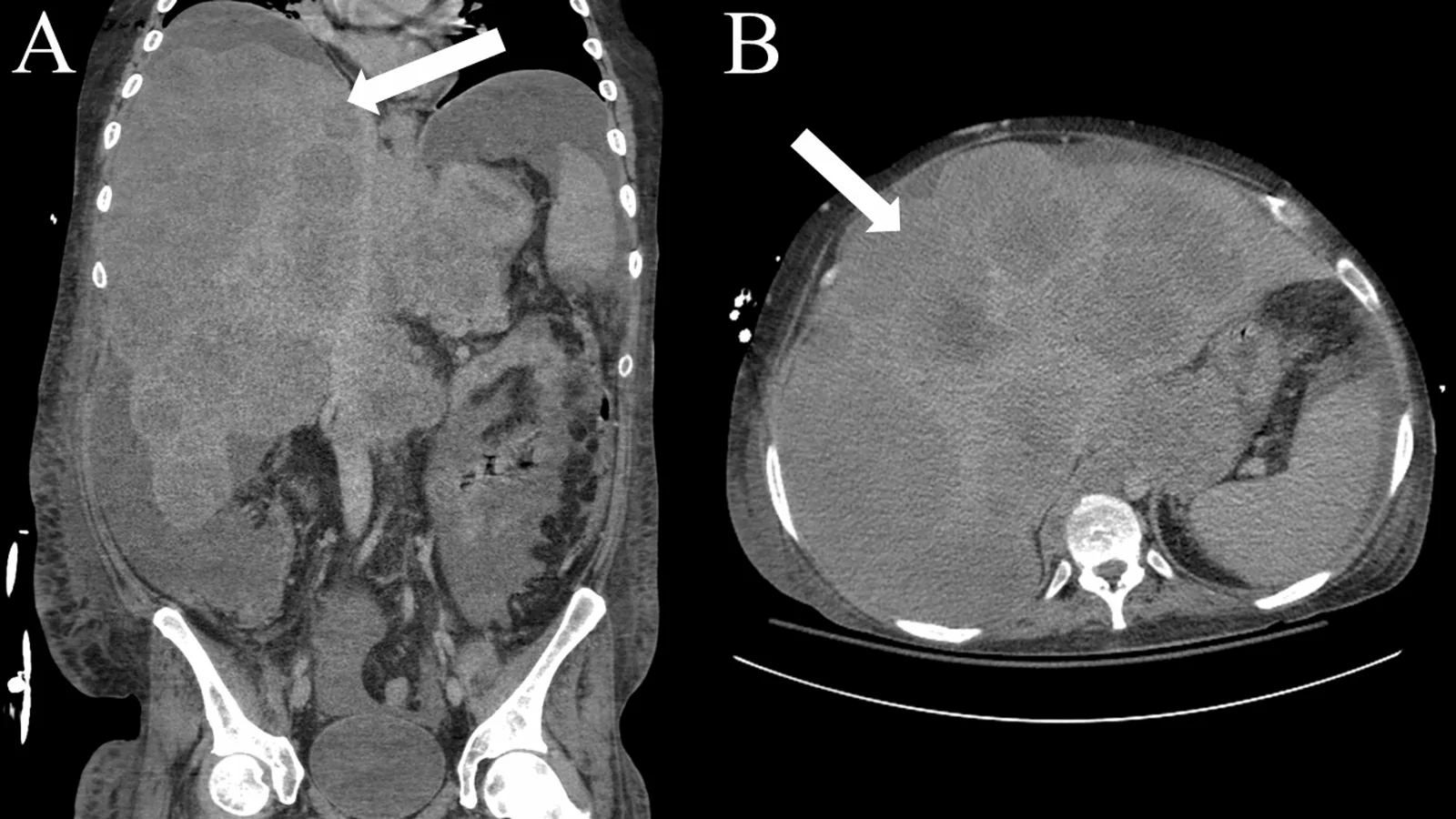
Contrast-enhanced CT of the abdomen and pelvis demonstrating marked hepatomegaly with extensive, coalescent hypoattenuating hepatic metastases (arrows), shown in coronal (A) and axial (B) views CT: computed tomography

Laboratory evaluation demonstrated severe metabolic, renal, and hepatic abnormalities, including marked elevations in uric acid, LDH, and lactate (Table 1).

**Table 1:** Pertinent laboratory findings on admission LDH: lactate dehydrogenase

Laboratory test	Patient’s results	Normal range	Units of measurement
Sodium	136	135-145	mmol/L
Potassium	7.6	3.6-5.2	mmol/L
Uric acid	18.1	4.0-8.5	mg/dL
Phosphorus	7.8	2.5-4.5	mg/dL
Calcium	7.8	8.6-10.5	mg/dL
Ionized calcium	1.03	1.13-1.32	mmol/L
Creatinine	1.67	0.5-1.0	mg/dL
Albumin	2.0	3.5-5.5	g/dL
Alanine aminotransferase	314	8-40	U/L
Aspartate aminotransferase	810	8-40	U/L
Alkaline phosphatase	2,058	30-100	U/L
Total bilirubin	8.2	0.1-1.2	mg/dL
Bicarbonate	11	22-29	mmol/L
Lactate	14.4	0.5-2.2	mmol/L
LDH	3,543	94-250	U/L
White blood cell count	39.4	4.0-11.0	k/µL
Hemoglobin	12.1	13.5-17.5	g/dL
Platelets	192	150-400	k/µL

The patient met the laboratory thresholds for TLS according to the Cairo-Bishop classification on the basis of hyperuricemia, hyperkalemia, and hyperphosphatemia; although corrected calcium did not reach the threshold, ionized calcium was low at 1.03 mmol/L, consistent with true hypocalcemia. The serum creatinine of 1.67 mg/dL exceeded 1.5 times the upper limit of normal, meeting criteria for clinical TLS and corresponding to Grade II severity according to the Cairo-Bishop classification [2]. The patient was anuric on presentation and remained so throughout hospitalization. Notably, the presentation occurred approximately 17 days after completion of the continuous fluorouracil infusion, outside the conventional Cairo-Bishop treatment-related timeframe [2]. Given this delayed interval, progressive high-burden malignancy, and absence of a closer temporal relationship to systemic therapy, the presentation was considered most consistent with STLS.

The patient was managed in the ICU with vasopressor support, IV antibiotics for MSSA bacteremia, and medical management of hyperkalemia and metabolic acidosis. Rasburicase (3 mg IV) was administered upon recognition of TLS. Electrocardiography demonstrated peaked T waves in the setting of severe hyperkalemia, prompting immediate treatment with IV calcium, insulin and dextrose, and nebulized albuterol.

Renal replacement therapy was considered because of the patient's persistent anuria and metabolic derangements; however, this was considered to be nonbeneficial following multidisciplinary discussions regarding overall prognosis.

Given radiographic disease progression despite systemic therapy, extensive metastatic disease burden, poor functional status, and multiorgan failure, discussion with the family resulted in a transition to comfort-focused care. The patient was discharged to home hospice with ongoing vasopressor support for transport and died comfortably at home with family.

## Discussion

This case represents STLS in metastatic esophageal adenocarcinoma, a rare manifestation of an already uncommon phenomenon. While TLS is well described in hematologic malignancies, its occurrence in solid tumors is infrequent, and STLS in gastrointestinal adenocarcinomas is particularly rare [3,4].

A review of the literature demonstrates that STLS in gastroesophageal malignancies is exceedingly rare. Only a few isolated cases have been documented, primarily in gastric adenocarcinomas, with even fewer reported in esophageal cancer [4,5]. An early case described by Woo et al. reported STLS in a young patient with advanced gastric cancer and multiple hepatic metastases who presented prior to cytoreductive therapy [10]. Watanabe et al. reported a case of recurrent STLS in a patient with esophageal cancer who recovered following chemotherapy adjustments [5]. More recently, Prasad et al. described STLS in a patient with metastatic gastric adenocarcinoma, highlighting the grave prognosis [4]. Across reported cases of STLS in solid tumors, hepatic involvement has been associated with increased mortality [8,11]. This association may reflect the high purine burden and mechanical compression caused by extensive hepatic metastases, which can contribute to hepatic synthetic dysfunction and impaired uric acid metabolism [8]. Our patient’s presentation aligned with these findings, given his extensive hepatic metastases and rapid clinical deterioration.

Our patient had multiple risk factors predisposing to STLS, including massive tumor burden with extensive hepatic metastases, markedly elevated LDH, progressive disease refractory to therapy, and hypovolemia. Importantly, his last chemotherapy was administered more than two weeks before presentation, outside the conventional timeframe associated with treatment-related TLS, supporting a diagnosis of spontaneous TLS. This case illustrates that in treatment-refractory malignancy, ongoing tumor turnover and high disease burden may result in spontaneous tumor lysis independent of recent cytotoxic exposure.

The pathophysiology of TLS explains the severity of this presentation. Rapid tumor cell lysis leads to the release of potassium, phosphate, and nucleic acids. Uric acid accumulation can precipitate within renal tubules, causing obstructive nephropathy and acute kidney injury. Hyperkalemia can lead to life-threatening cardiac arrhythmias, while hyperphosphatemia results in calcium phosphate deposition and secondary hypocalcemia, which may cause seizures and neuromuscular instability [1].

This case also highlights the diagnostic challenge of TLS in critically ill oncology patients. The etiology of acute kidney injury in this critically ill patient was likely multifactorial. Concurrent MSSA septic shock with hypotension, poor oral intake, recent large-volume paracentesis, and hepatic failure could all have contributed to renal hypoperfusion and kidney injury. However, these competing etiologies did not fully account for the characteristic constellation of marked hyperuricemia, hyperkalemia, and hyperphosphatemia occurring in the setting of massive tumor burden. These simultaneous abnormalities fulfilled laboratory TLS criteria and supported TLS as an important contributor to the acute kidney injury, while the concurrent critical illnesses likely contributed to the severity of his overall renal and multiorgan dysfunction.

Early recognition of TLS is essential, as timely intervention with hydration, electrolyte correction, and uric acid-lowering therapy may prevent irreversible organ damage [1]. During periods of greatest risk or active metabolic derangement, laboratory assessment may be required every 6-12 hours, with frequency subsequently individualized according to the severity and trajectory of abnormalities [12]. However, in patients with advanced malignancy and multiorgan failure, management must be aligned with overall prognosis and goals of care.

## Conclusions

STLS is a rare but life-threatening complication in patients with advanced solid tumors, including gastroesophageal adenocarcinoma. This case underscores the importance of considering TLS in the differential diagnosis of critically ill oncology patients despite variable primary malignancies. It also highlights that TLS can occur outside the traditional treatment-related window and may present concurrently with other critical illnesses, such as septic shock, making diagnosis challenging. Recognition of the characteristic metabolic abnormalities and application of the Cairo-Bishop criteria remain essential for timely identification. Clinicians should maintain a high index of suspicion for TLS in patients with high tumor burden, progressive disease, and acute renal failure, even in the absence of recent chemotherapy. Early recognition is critical, as TLS can rapidly lead to life-threatening complications, including arrhythmias, severe renal failure, and multiorgan dysfunction. Timely intervention with hydration, electrolyte correction, and uric acid-lowering therapy may prevent irreversible organ damage. However, in patients with advanced malignancy and multiorgan failure, management must be aligned with overall prognosis and goals of care.
